# Sequence similarity network and protein structure prediction offer insights into the evolution of microbial pathways for ferrous iron oxidation

**DOI:** 10.1128/msystems.00720-23

**Published:** 2023-09-28

**Authors:** Liangzhi Li, Zhenghua Liu, Delong Meng, Yongjun Liu, Tianbo Liu, Chengying Jiang, Huaqun Yin

**Affiliations:** 1 School of Minerals Processing and Bioengineering, Central South University, Changsha, China; 2 Key Laboratory of Biometallurgy of Ministry of Education, Central South University, Changsha, China; 3 Hunan Tobacco Science Institute, Changsha, China; 4 University of Chinese Academy of Sciences, Beijing, China; 5 State Key Laboratory of Microbial Resources, Institute of Microbiology, Chinese Academy of Sciences, Beijing, China; University of Pretoria, Hatfield, Pretoria, South Africa

**Keywords:** ferrous iron oxidation, horizontal gene transfer, sequence similarity network, protein structure prediction

## Abstract

Dissimilatory ferrous iron [Fe(II)] oxidation is a well-established microbial energy generation strategy. This study aims to comprehensively investigate the distribution and evolution of recognized Fe(II) oxidation pathways through comparative analysis. Interestingly, we have discovered a wide range of taxonomic groups that harbor homologs to known Fe(II) oxidation proteins. The presence of these homologs among phylogenetically distant lineages and their frequent association with mobile genetic elements strongly suggest horizontal gene transfer events involving Fe(II) oxidation proteins, such as the *rus* operon of *Acidithiobacillus* and Cyc572 from *Leptospirillum* lineages belonging to classes *Gammaproteobacteria* and *Betaproteobacteria* often present at the hub positions of the protein sequence similarity networks from which homologs of other taxa are derived. In addition, RoseTTAFold predictions have provided valuable insights into the structural characteristics of previously unknown Fe(II) oxidation components. Despite having limited sequence identity, a significant number of acknowledged proteins involved in different Fe(II) oxidation pathways exhibit close structural similarities, including Cyc2 and Cyc572. Collectively, this study significantly enhances our understanding of the distribution and evolution of microbial ferrous iron oxidation pathways.

**IMPORTANCE:**

Microbial Fe(II) oxidation is a crucial process that harnesses and converts the energy available in Fe, contributing significantly to global element cycling. However, there are still many aspects of this process that remain unexplored. In this study, we utilized a combination of comparative genomics, sequence similarity network analysis, and artificial intelligence-driven structure modeling methods to address the lack of structural information on Fe(II) oxidation proteins and offer a comprehensive perspective on the evolution of Fe(II) oxidation pathways. Our findings suggest that several microbial Fe(II) oxidation pathways currently known may have originated within classes *Gammaproteobacteria* and *Betaproteobacteria*.

## INTRODUCTION

Iron is the fourth most abundant element in the Earth’s crust, with an estimated abundance of around 5% (1). The ability to perform dissimilatory ferrous iron [Fe(II)] oxidation has been discovered and characterized in various lineages of Bacteria and Archaea. This process has had a significant impact on global biogeochemical cycling processes, including the formation and dissolution of iron ore. The switch between the two most common iron redox states, ferrous iron [Fe(II)] and ferric iron [Fe(III)], transfers just one electron. Despite this simplicity, it has become one of the oldest and most widespread energy-producing strategies. In fact, there is a hypothesis that the Fe(II)-oxidizing chemoautotrophic microbes may have played a key role in the formation of banded iron formations, which are the most prominent sedimentary iron mineral deposits on Earth and were formed during the late Archean and early Proterozoic Eon (2).

Research on microbial Fe(II) oxidation has primarily focused on acidophilic microorganisms, such as *Acidithiobacillus* (*At.*) *ferrooxidans* (3) and *Leptospirillum (L.) ferrooxidans* (4), that rely on the oxidation of iron minerals, such as ferrous sulfide (FeS) and pyrite (FeS_2_), for energy and growth. In acidic environments like acid mine drainage, oxygen (O_2_) serves as the most abundant and bioenergetically favorable terminal electron acceptor for Fe(II) oxidation by these microorganisms (5, 6). However, certain neutrophilic bacterial lineages, such as *Sideroxydans (S.) lithotrophicus* (7) and *Mariprofundus (M.) ferrooxydans* (8), have evolved the capability to carry out oxygen-dependent Fe(II) oxidation at the iron-rich microoxic interface through specialized electron transport chains, even at circumneutral pH. These Fe(II) oxidation processes generate ATP and NAD(H), which are essential for sustaining microbial metabolism, including carbon assimilation and biosynthesis.

There are currently at least eight distinct microbial pathways/models for Fe(II) oxidation that have been deciphered (summarized in Table 1). Additionally, an increasing number of studies are focused on identifying novel genes or gene clusters involved in Fe(II) oxidation and validating the iron oxidation capabilities conferred by homologs of these pathways within a wider range of taxa (9 – 11).

**TABLE 1:** Canonical Fe(II) oxidation pathways in different microbial groups

Type	Species	Protein	Role/function	Reference
Acidophilic Fe(II)-oxidizing bacterium	*Acidithiobacillus* spp.	Cyc2	Outer membrane porin-cytochrome protein	(12 – 15)
		Cyc1	Cytochrome *c_4_* protein	
		Rus	Rusticyanin, electron transmitter	
		Cup	Copper-containing protein, electron transmitter	
		CoxBACD	Cytochrome *aa_3_* oxidase complex	
		CtaA	Heme A synthase	
		CtaB	Heme O synthase	
		CycA	Diheme cytochrome *c_4_* protein	
		PetABC	*bc_1_* complex	
		SdrA	Accessory NADH complex subunit	
		Iro	Iron oxidase, high-potential iron-sulfur protein	
	*Leptospirillum* spp.	Cyc572	Outer membrane cytochrome *c*	(16 – 18)
		Cyc579	Periplasmic cytochrome *c*	
		Cyt *bc_1_*	Cytochrome *bc_1_*	
		Cyt *cbb_3_*	*cbb_3_*-type cytochrome *c* oxidase	
Neutrophilic Fe(II)-oxidizing bacterium	*Rhodopseudomonas* spp.	PioA	Decaheme cytochrome *c*	(19 – 21)
		PioB	Outer membrane protein	
		PioC	High-potential iron-sulfur protein	
	*Sideroxydans lithotrophicus*	MtoA	Decaheme cytochrome *c*	(7, 22 – 25)
		MtoB	Outer membrane protein	
		MtoD	Cytochrome *c* protein	
		CymA	Tetraheme cytochrome *c* protein	
	*Rhodobacter* spp.	FoxE	Bihemic cytochrome *c* protein	(26)
		FoxY	Pyrroloquinoline quinone containing periplasmic protein	
		FoxZ	Inner membrane transport protein	
	*Mariprofundus ferrooxydans*	Mob	Periplasmic Fe–S molybdopterin oxidoreductase	(8, 27, 28)
		Cyc2-PV1	Cyc2-like iron oxidase	
Acidophilic Fe(II)-oxidizing archaeon	*Metallosphaera* spp.	FoxA	Putative heme copper oxidase subunit	(29 – 31)
		FoxB	Putative heme copper oxidase subunit	
		FoxC	Putative cytochrome *c* oxidase	
		FoxD	Putative cytochrome *c* oxidase	
		Mco	Multi-copper oxidase	
	*Ferroplasma* spp.	Sulfocyanin	Primary electron acceptor from Fe(II)	(32)
		Cyt *caa_3_*	*caa_3_*-type cytochrome c oxidase	

Although the pathways of Fe(II) oxidation vary significantly among different iron oxidizers, there are certain similarities in their overall organizational patterns (33):

1. Oxidation of Fe(II) occurs either in the periplasmic space or at the outer membrane.
2. The abundance of functional redox proteins is relatively high (especially in Gram-negative bacteria), even though the difference in redox potential between Fe(II)/Fe(III) and O_2_/H_2_O is small.
3. Cytochromes generally act as the Fe(II) oxidase. *c*-Type cytochromes, as the main Fe(II) oxidases, contain a heme group that acts as a prosthetic group for electron transport (C-Cyt) (34). They are typically found on the outer membrane of bacteria. These enzymes exhibit a high affinity for Fe(II) and function as electron carriers, shuttling electrons from Fe(II) to an electron acceptor, such as molecular oxygen or other terminal electron acceptors, thus driving the oxidation reaction as a whole. The Fe(II) oxidation process mediated by *c*-type cytochromes consists of multiple several steps (35). Initially, the cytochrome binds to the Fe(II) ion, facilitating the transfer of electrons from Fe(II) to the heme group within the cytochrome. Binding of Fe(II) induces a conformational change in the cytochrome, enabling interaction with other enzymes or proteins involved in electron transfer. As a result, the electrons are transferred through the electron transport chain, facilitating the reduction of electron acceptors and the generation of energy for microbial growth. Additionally, the oxidase activity of *c*-type cytochromes can be regulated by various factors, such as oxygen concentration, pH, and the presence of other redox-active metals (35 – 38). These enzymes display diverse structural and functional properties, enabling microbial communities to adapt to various environmental conditions.
4. The transfer of electrons from Fe(II) to the cytoplasmic membrane is facilitated by vertically organized electron shuttles that connect the external medium with the cytoplasm. These shuttles are distinct from the laterally arranged redox proteins found in most respiratory chains.

The vertical organization of the iron respiratory chain components offers several advantages (33):

1. It prevents the precipitation of Fe(III) in the cytoplasm due to its neutral pH by sequestering iron outside the cell.
2. It separates the respiratory chain from oxidative stress caused by the Fenton reaction.
3. It helps maintain a neutral pH in the cytoplasm, which is essential for the survival of acidophiles, by consuming protons through the reduction of O_2_ to H_2_O.

An example from the best-studied case of the model microorganism *At. ferrooxidans* (13) highlights these processes: electrons are initially extracted from extracellular Fe(II) by the outer membrane cytochrome *c* Cyc2 and then transferred to the periplasmic blue copper protein rusticyanin. From the “branch point” protein rusticyanin, the electrons can then flow either downstream, reducing O_2_ to water via cytochrome *c_4_* Cyc1 and the *aa_3_*-type cytochrome oxidase complex, or upstream, utilizing the proton motive force across the inner membrane to overcome the unfavorable thermodynamic gradient and transfer electrons to the NADH1 complex, bypassing the cytochrome *c_4_* CycA1, the *bc_1_* complex and membrane-associated quinones. Higher redox potentials are observed at acidic pH levels than at neutral pH conditions, wherein both Fe(II)/Fe(III) species remain soluble. For example, in the presence of sulfate, which is the most common complexing agent in acid mine/rock drainage waters, the redox potential at pH 3 can reach approximately +0.720 V (5).

Previous studies have primarily investigated individual components of particular Fe(II) oxidation models or specific details within the Fe(II) oxidation pathway of certain species. Consequently, there is currently a deficiency in our overall understanding of the distribution and progression of these fascinating Fe(II) oxidation pathways. Furthermore, although various Fe(II) oxidizers have been proposed, our comprehension of their molecular-level Fe(II) oxidation pathways remains significantly lacking. Meanwhile, several Fe(II) oxidation components lack structure information, impeding further investigations into their molecular mechanisms.

We aimed to provide a comprehensive overview of the distribution and evolution of experimentally characterized microbial ferrous iron oxidation pathways. We achieved this objective by employing sequence similarity network (SSN) analysis, phylogenetic analysis, and genomic comparisons to offer a holistic perspective. Additionally, we utilized RoseTTAFold (39), an artificial intelligence (AI)-driven method, to generate high-quality three-dimensional (3D) structures of canonical proteins involved in microbial Fe(II) oxidation. These proteins have been under-studied and lack available structural data. Subsequently, we conducted fold recognition/comparison, conservation analysis, and electron hopping pathway prediction to gain insights into their mechanisms. The SSN analysis is a computational approach used to investigate the relationships between sequences based on their similarity (40). Unlike traditional phylogenetic tree construction, SSN analysis clusters sequences into groups called "network communities" based on sequence similarity, allowing for the detection of potential evolutionary relationships even among distantly related organisms (41). In our study, we employed the SSN analysis to explore the clustering patterns of proteins involved in iron oxidation across different organisms. By visualizing the network communities, we aimed to identify potential horizontal gene transfer (HGT) events or evolutionary connections that might have shaped the distribution and evolution of these iron metabolic cytochromes (42).

## RESULTS

### The overall models of Fe(II) oxidation

**Fig 1:**
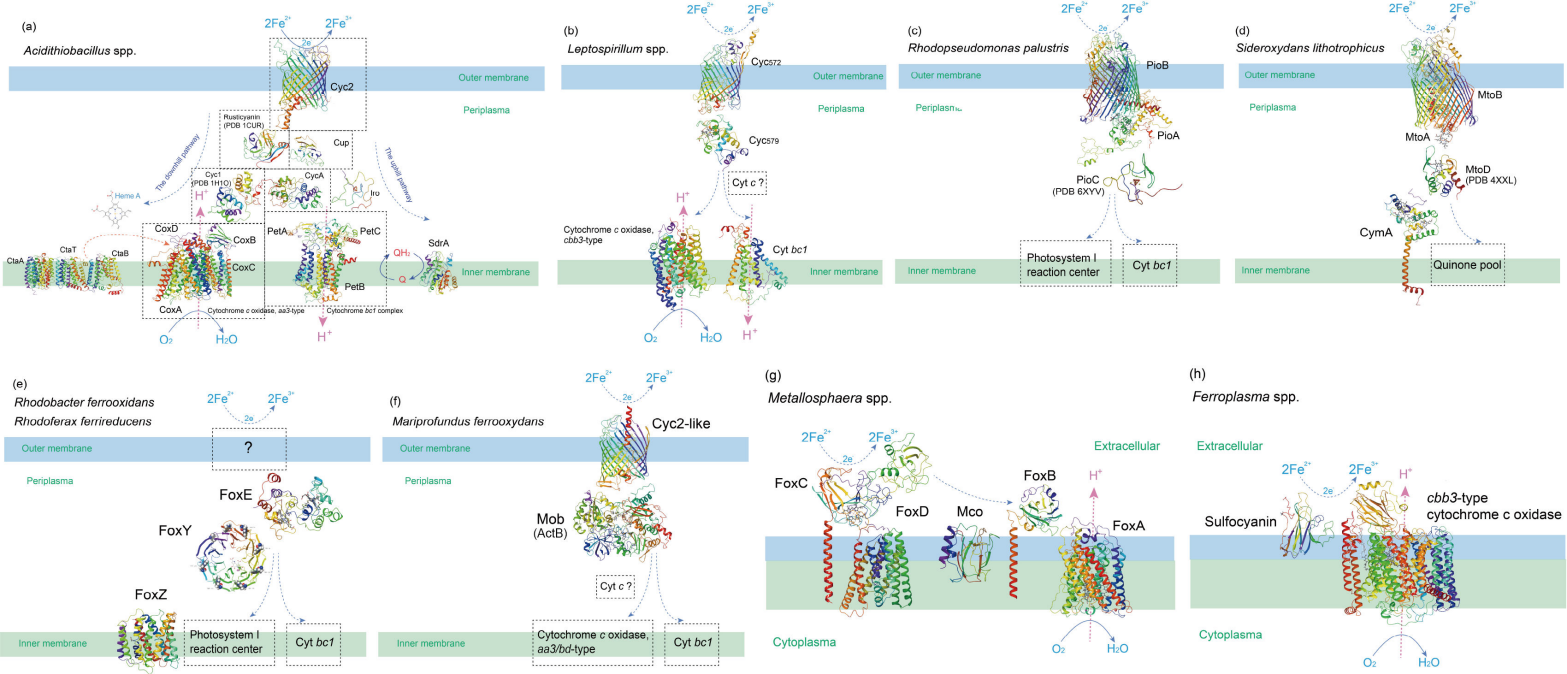
Models with protein structures predicted by RoseTTAFold showing the roles and arrangements of different Fe(II) oxidation proteins from various microbial groups as indicated in respective previous studies: (a) *Acidithiobacillus* spp.; (b) *Leptospirillum* spp.; (c) *Rhodopseudomonas palustris*; (d) *Sideroxydans lithotrophicus*; (e) *Rhodobacter ferrooxidans* and *Rhodoferax ferrireducens*; (f) *Mariprofundus ferrooxydans*; (g) *Metallosphaera* spp.; (h) *Ferroplasma* spp. The overall fold of the polypeptide chain is ramp-colored from red (N-terminal) to purple (C-terminal).

We first utilized FeGenie (43) to identify candidate genes associated with iron oxidation in the genomes of reported iron oxidizer. From the FeGenie results, we obtained the representative sequences of known Fe(II) oxidation proteins as the initial query sequences for subsequent analyses (see Supplementary Material at https://doi.org/10.6084/m9.figshare.23652387.v1). We predicted the structure of Fe(II) oxidation proteins with unknown 3D structures using RoseTTAFold (39), as exhibited and discussed in detail in the following sections. We then constructed models that demonstrate the roles and arrangements of various Fe(II) oxidation proteins, as indicated in previous studies. These models offer a comprehensive view of the diverse Fe(II) oxidation pathways (Fig. 1). It is important to note that the predictions of RoseTTAFold (39), which are shown below, are primarily *de novo*. This means that they do not rely on any template that shares more than 30% identity with the query or covers more than 35% of the sequence. The structural predictions provide valuable insights that can help fill the gaps in our understanding of the functional roles and molecular details of these proteins in the whole Fe(II) oxidation-related system.

### The *rus* and *pet* operons

**Fig 2:**
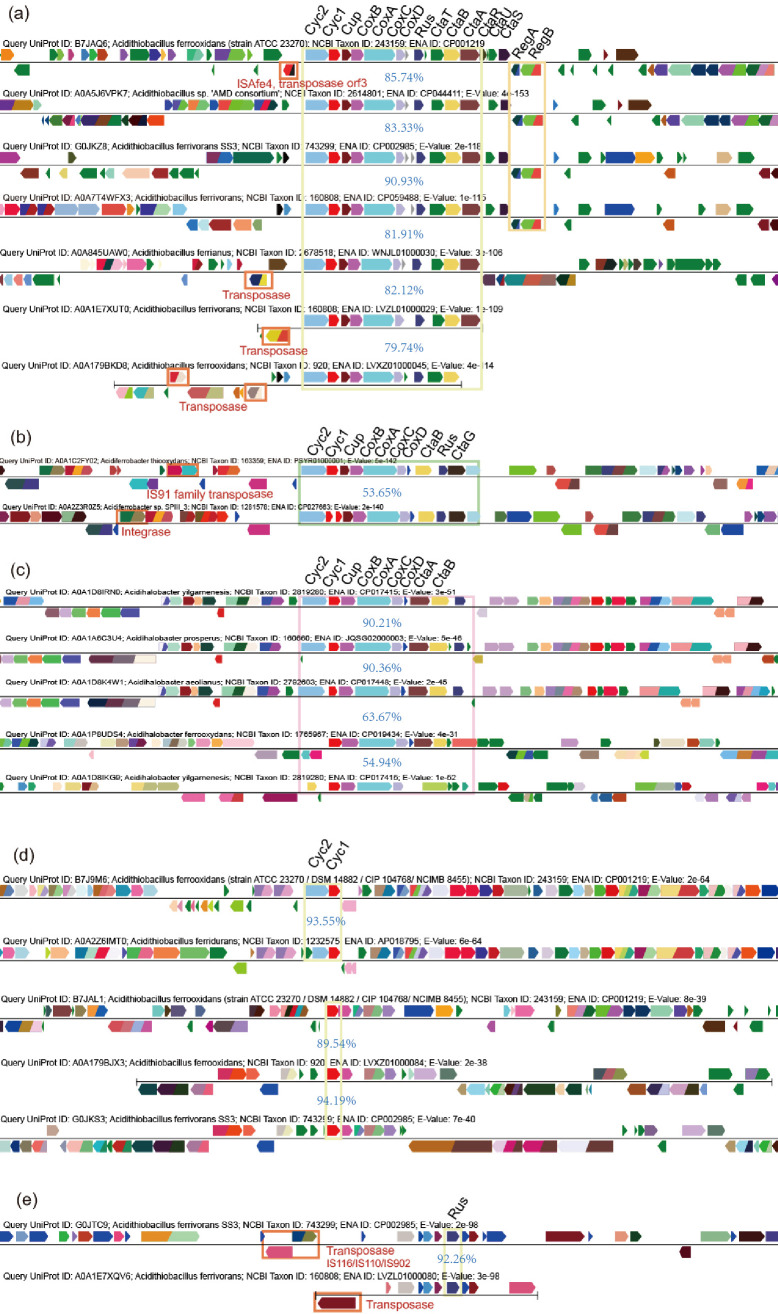
Comparison of operon organization and genome context of *rus* operon components. (a) The *rus* operon in organization of *cyc2-cyc1-cup-coxB-coxA-coxC-coxD-rus-ctaT-ctaB-ctaA* in *Acidithiobacillus* spp. (b) Similar *rus* operons present in *Acidiferrobacter* (in organization of *cyc2-cyc1-cup-coxB-coxA-coxC-coxD-ctaB-rus-ctaG*) and (c) similar *rus* operons present in *Acidihalobacter* spp. (in organization of *cyc2-cyc1-cup-coxB-coxA-coxC-coxD-ctaA-ctaB-rus*). (d) Stand-alone genes of *cyc2* and *cyc1* identified in *Acidithiobacillus*. (e) Stand-alone genes of *rus* identified in *Acidithiobacillus*. Transposases and integrases are found in the flanking regions of these *rus* operons in respective genomes (marked with red rectangles). The color of each gene is assigned based on domain classification (with the same domains showing the same color). Average amino acid identities (AAIs) of the genes within the operons are indicated in blue.

The pathway of Fe(II) oxidation in *Acidithiobacillus* spp. is predominantly regulated by the *rus* and *pet* operons (13, 15). Counterparts of these operons are also identified in *Acidiferrobacter* and *Acidihalobacter,* as illustrated in Fig. 2.

Specifically, we have observed the conservation of the *rus* operon in *Acidithiobacillus* spp. (class *Acidithiobacillia*), with an organization of *cyc2-cyc1-cup-coxB-coxA-coxC-coxD-rus-ctaT-ctaB-ctaA*. Interestingly, we have also found similar but slightly different *rus* operons in *Acidiferrobacter* spp. (class *Gammaproteobacteria*) and *Acidihalobacter* spp. (class *Betaproteobacteria*). In *Acidiferrobacter* spp., the organization is *cyc2-cyc1-cup-coxB-coxA-coxC-coxD-ctaB-rus-ctaG*, and in *Acidihalobacter* spp., it is *cyc2-cyc1-cup-coxB-coxA-coxC-coxD-ctaA-ctaB-rus* (Fig. 2). These findings suggest cross-class evolutionary associations. Moreover, the flanking regions of these *rus* operons in the respective genomes are variable and contain mobile genetic elements (MGEs), such as transposases and integrases (Fig. 2). Additionally, we have identified stand-alone genes of *cyc2, cyc1*, and *rus* in *Acidithiobacillus* spp., with some cases involving flanking transposases (Fig. 2).

**Fig 3:**
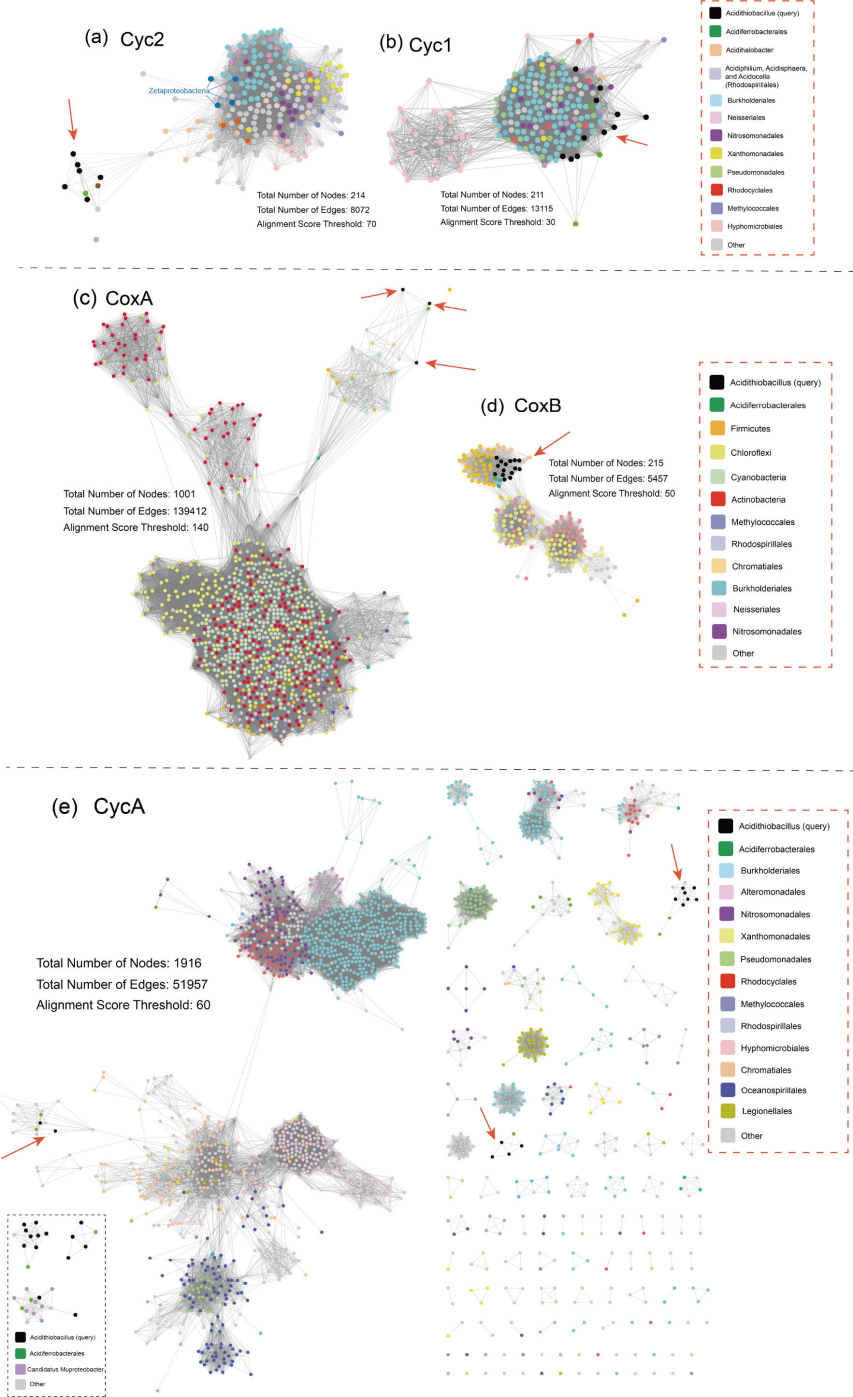
Protein SSNs of representative proteins encoded by the Fe(II) oxidation operons of *Acidithiobacillus* for the visualization of the distribution and evolution diagram of these proteins and their putative homologs: (a) Cyc2, (b) Cyc1, (c) CoxA, (d) CoxB, and (e) CycA. The total number of nodes/edges and alignment score threshold (corresponding to 35% sequence identity) for final SSN construction are shown. The sub-SSN showing the first neighbors of the query sequence is marked with a black dotted rectangle frame.

We further conduct SSN analyses on individual genes in the *rus* operon to visualize the distribution and evolution diagram of these genes and their putative homologs (Fig. 3). The first gene of the *rus* operon of *Acidithiobacillus ferrooxidans* encodes the cytochrome *c* protein AfCyc2, which serves as the initial electron acceptor located on the outer membrane. The second gene encodes a cytochrome *c_4_* protein (AfCyc1), which associates with rusticyanin (Rus) and a copper-containing protein (Cup) to transfer electrons from AfCyc2 to the cytochrome *aa_3_* oxidase complex (CoxBACD).

The SSN diagram (see Fig. 3a) reveals that *Acidithiobacillus*’ Cyc2 proteins are connected to a dense cluster consisting mainly of homologs from *Rhodanobacteraceae* (*Gammaproteobacteria*), *Hyphomicrobiales* (*Alphaproteobacteria*), *Burkholderiales* (*Betaproteobacteria*), *Chromobacteriaceae* (*Neisseriales; Betaproteobacteria*), and *Nitrosomonadales* (*Betaproteobacteria*), as well as reported typical acid mine drainage (AMD) heterotrophs of *Rhodospirillales, Alphaproteobacteria* (i.e., *Acidiphilium, Acidisphaera*, and *Acidocella*) (44). Similarly, the SSN of AfCyc1 (see Fig. 3b) also shows the presence of these lineages. However, the *cox* cluster (CoxA and CoxB) of *Acidithiobacillus* appears to have derived from distant lineages such as those belonging to the phyla *Chloroflexi, Actinobacteria, Firmicutes*, and *Cyanobacteria* (Fig. 3c and d). Meanwhile, Rus and Cup cluster with *Acidiferrobacter, Acidihalobacter*, and *Bacillales*, including *Alicyclobacillus*, in the reconstructed phylogeny (see Fig. S1 and S2 at https://doi.org/10.6084/m9.figshare.20079053.v4). The clustering with phylogenetically distant lineages suggests the occurrences of HGT on the Fe(II) oxidation genes.

Adjacent to the *cox* cluster in microbial iron oxidizer genomes are genes that participate in heme biosynthesis (*ctaAB*), the cofactor essential for cytochrome *aa_3_* oxidase (Fig. 2). SSN analysis revealed that homologs of CtaA and CtaB (heme A/O synthases) are widely distributed in lineages from *Proteobacteria*, *Terrabacteria* group, and even *Eukaryota*. From the sub-SSN illustrating the immediate neighbors of AfCtaA and AfCtaB, it is observed that AfCtaA and AfCtaB are closely related to homologs from *Alphaproteobacteria* lineages, including *Hyphomicrobiales*, *Rhodospirillales*, and *Sphingomonadales*. This suggests the occurrence of cross-class HGT (see Fig. S3 at https://doi.org/10.6084/m9.figshare.20079053.v4, dotted black frame).

The periplasmic proteins rusticyanin and AfCyc1 have previously been crystalized and characterized (45). According to RoseTTAFold, the structure of the undetermined protein AfCyc2, predicted to be a β-barrel porin-cytochrome fusion protein, aligns most closely with Protein Data Bank (PDB) entry 5O78 (phosphoporin phoE), with a TM-score of 0.80, root-mean-square deviation (RMSD) of 3.4 Å, and sequence identity of 10% when queried with the Dali server (46). This finding is consistent with a prior investigation (47).

In *Acidithiobacillus* and *Acidiferrobacter,* the *pet* operon responsible for Fe(II) oxidation-related operon is organized as *cycA-sdrA-petA-petB-petC* (see Fig. S4 at https://doi.org/10.6084/m9.figshare.20079053.v4). The first gene of the *pet* operon, *cycA*, encodes the diheme cytochrome *c_4_* protein AfCycA, responsible for the transfer of electron from rusticyanin to complex *bc_1_* (PetABC). The gene *petB* encodes cytochrome *b*, *petA* encodes an iron-sulfur protein Rieske, and *petC* encodes cytochrome *c_1_*. The second gene of the *pet* operon, *sdrA,* encodes a putative accessory NADH complex subunit. The sub-SSN of the first neighbors of AfCycA (Fig. 3e, dotted black frame) reveals that AfCycA is clustering with homologs from *Acidiferrobacterales* (*Gammaproteobacteria*) and Candidatus *Muproteobacter* (unclassified *Proteobacteria*). They are all derived from a hub-like cluster that mainly consists of homologs from *Chromatiales* (*Gammaproteobacteria*) (Fig. 3e). The SSNs of SdrA, PetA, PetB, and PetC also frequently include taxa such as *Xanthomonadales* (*Gammaproteobacteria*), *Thiotrichales* (*Gammaproteobacteria*), *Burkholderiales* (*Betaproteobacteria*), *Nitrosomonadales* (*Betaproteobacteria*), *Hyphomicrobiales* (*Alphaproteobacteria*), and *Oceanospirillales* (*Gammaproteobacteria*) (see Fig. S5 at https://doi.org/10.6084/m9.figshare.20079053.v4). Additionally, in several strains of *At. ferrooxidans* and *At. ferrianus*, we observed conserved genes located downstream of the *pet* operon, such as the *iro-cytb/b6* cluster encoding iron oxidase (Iro, a high-potential iron-sulfur protein) and cytochrome *b/b_6_*, or the *res* operon that encodes molecular chaperones ResB and ResC that facilitate maturation of cytochromes *c_4_* and *c_1_*, respectively (see Fig. S4 at https://doi.org/10.6084/m9.figshare.20079053.v4). Analysis of SSNs for Iro, ResB, and ResC demonstrates the presence of similar adjacent taxa to that of PetABC (see Fig. S6 at https://doi.org/10.6084/m9.figshare.20079053.v4). Although *Acidithiobacillus* as we know is firstly and well clarified to utilize the genes in operons *rus* and *pet* for iron oxidation (48, 49), our SSN results indicate that these genes from *Acidithiobacillus* spp. might not be ancient or original, as they do not occupy a central position in the SSNs. Instead, they appear on the periphery of respective SSNs and cluster with phylogenetically distant lineages. Furthermore, the gene operons *rus* and *pet* display variable genome context, abundant flanking MGEs, and deviated G + C content against corresponding genomes (Fig. 2; see Fig. S4 and S7 at https://doi.org/10.6084/m9.figshare.20079053.v4). These pieces of evidence suggest that HGT events may have promoted the acquisitions of the iron oxidation ability in *Acidithiobacillus* spp.

### The Cyc572–Cyc579 system

**Fig 4:**
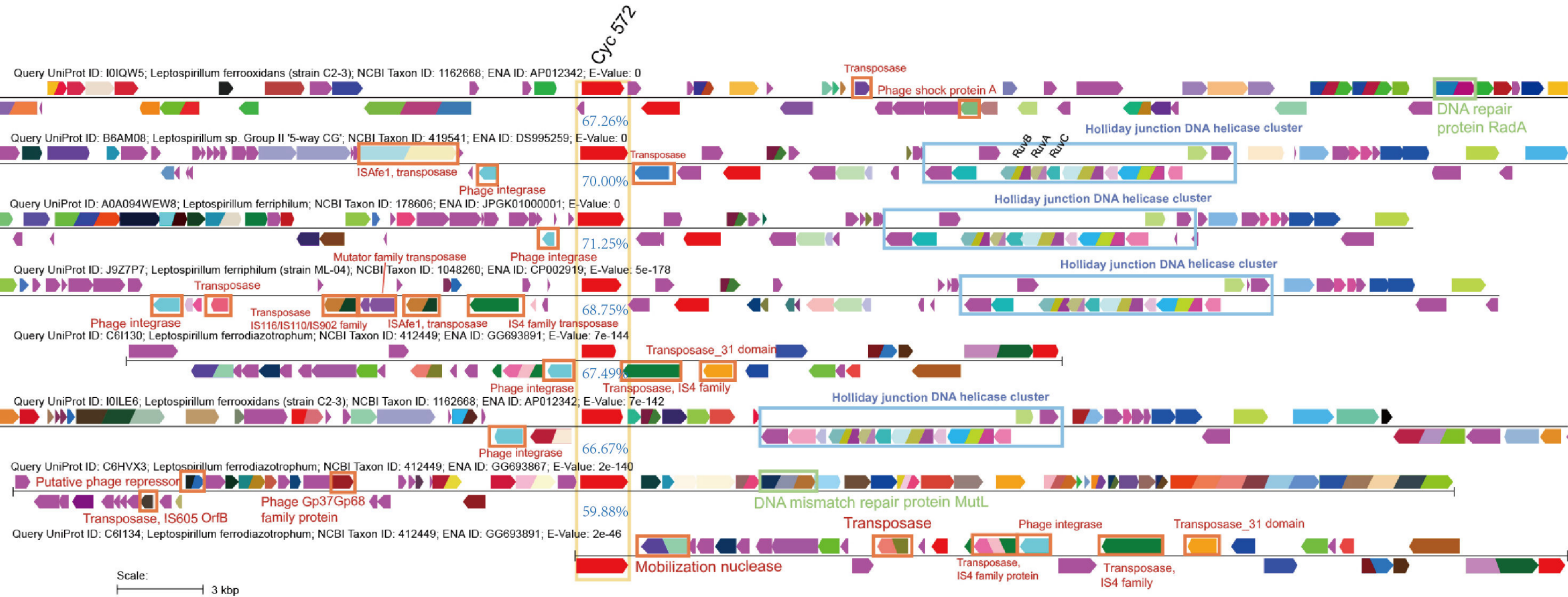
Genome context comparisons of Cyc572 from *Leptospirillum* spp. as a typical case of Fe(II) oxidation gene with highly variable gene contexts and abundant flanking MGEs (e.g., transposase and phage integrase), as HGT indicators. The color of each gene is assigned based on domain classification (with the same domains showing the same color). Average AAIs of the genes within the operons are indicated in blue.

Another group of acidophilic and widely applied iron-oxidizing bacteria, known as *Leptospirillum* spp. (phylum *Nitrospirae*), has been found to utilize the outer membrane cytochrome *c* Cyc572 as a direct oxidant and first electron acceptor of Fe(II). Subsequently, they transfer electrons through the periplasmic cytochrome *c* Cyc579 and the cytochrome *bc_1_,* ultimately reaching a *cbb_3_*-type cytochrome *c* oxidase (Fig. 1b) (16 – 18). The genes encoding these components are not found to be clustered in the genomes of *Leptospirillum* spp. Interestingly, we have detected strong HGT signals of these iron metabolism genes, particularly the cytochrome *c* Cyc572 in *Leptospirillum* spp. Significant variability can be observed in the genome contexts of *Leptospirillum*'s Cyc572, particularly in terms of gene G+C content, when compared to their respective genomes (see Fig. 4; Fig. S7c at https://doi.org/10.6084/m9.figshare.20079053.v4). Moreover, numerous MGEs, including transposase and phage integrase, which are indicators of HGT, are found flanking the Cyc572 in *Leptospirillum* spp. These MGEs are present at an average of three to five per genome. Additionally, several mismatch-repair proteins that may modulate the efficiency of HGT are also found in this region (50) (Fig. 4). As illustrated in the SSN diagram, *Leptospirillum*’s Cyc572 is derived from a dense cluster containing homologs from *Acidobacteria* and *Nitrospira*, which are putative HGT donors (see Fig. S8a at https://doi.org/10.6084/m9.figshare.20079053.v4). The SSN diagram also reveals similar results for other iron metabolism genes of *Leptospirillum* spp., although their adjacent taxa appear to differ. For example, the taxa Candidatus *Brocadiales* (phylum *Planctomycetes*), *Desulfobacterota* (previous *Deltaproteobacteria*), and *Nitrospinales* (phylum *Nitrospinae*) are dominant in the first neighbors of *Leptospirillum*’s cytochrome *bc_1_*. On the other hand, *Leptospirillum*’s cytochrome *cbb_3_* oxidase is closely linking to homologs from cross-phylum lineages, including *Bacillales* (phylum *Firmicutes*) and *Burkholderiales* (phylum *Proteobacteria*) (see Fig. S8 at https://doi.org/10.6084/m9.figshare.20079053.v4). The predicted 3D model of Cyc572 has few close structural homologs when searched against the PDB database using DALI (46). It shows the highest alignment score with structures of PorB porin proteins (PDB entries 4AUI, 3WI4) with identities of about 10% and RMSDs of around 3.0 Å. PorB belongs to a group of channel-forming outer membrane proteins that uptake small solutes (51). Like PorB, Cyc572 is predicted to have a 16-stranded β-barrel topology with connecting turns and surface-exposed, inter-strand loops on the extracellular side. However, electrostatic surface potential analysis reveals strong electronegative charges on the surface of the funnel approaching the pore and the periplasmic periphery of Cyc572, in sharp contrast to the predominantly electropositive surface of PorB (see Fig. S9 at https://doi.org/10.6084/m9.figshare.20079053.v4). Interestingly, several key residues previously reported are also conserved in Cyc572 (see Fig. S9c at https://doi.org/10.6084/m9.figshare.20079053.v4, marked with green rectangles): Gly261 and Asp262 [corresponding to Gly103 and Asp104 of PorB from *Neisseria meningitidis* (PDB 3VZT)] that contribute to antibiotic resistance, and Arg216 and Arg300 (referring to Lys62 and Arg124 of PDB 3VZT), which support ligation with the adenosine moiety of ATP (52). Both LfCyc572 and AfCyc2 contain the conserved characteristic N-terminal heme-binding motif (C51-xx-C54-H55 in LfCyc572 order, see Fig. S9c at https://doi.org/10.6084/m9.figshare.20079053.v4, marked with blue rectangles), despite significant sequence divergence (11.4% sequence identity), which suggests that both of them are cytochrome-porin fusion proteins. Additionally, a set of aromatic amino acids with hydrophobic side chains are uniquely conserved among Cyc572 and PorB, such as Tyr108, Trp194, Tyr231, Tyr275, Phe444, Phe457, Tyr471, Phe482, Tyr543, and Phe553 (in Cyc572 order, see Fig. S9c at https://doi.org/10.6084/m9.figshare.20079053.v4, marked with red rectangles).

We found that the protein structure Cyc579 exhibits the closest similarity to the *Kuenenia stuttgartiensis* cytochrome *c*-552 (53) (PDB 5MXY) with RMSD 1.8 Å and 22.0% sequence identity, as determined by the highest TM-align score computed using the DALI server (see Fig. S10 at https://doi.org/10.6084/m9.figshare.20079053.v4). Cyc579 displays the canonical type I cytochrome *c* fold with the heme cofactor proximally coordinated by His47 and distally by Met91 resembling *K. stuttgartiensis* cytochrome *c*-552.

### The *pioABC* and *mtoABD-cymA* operons

**Fig 5:**
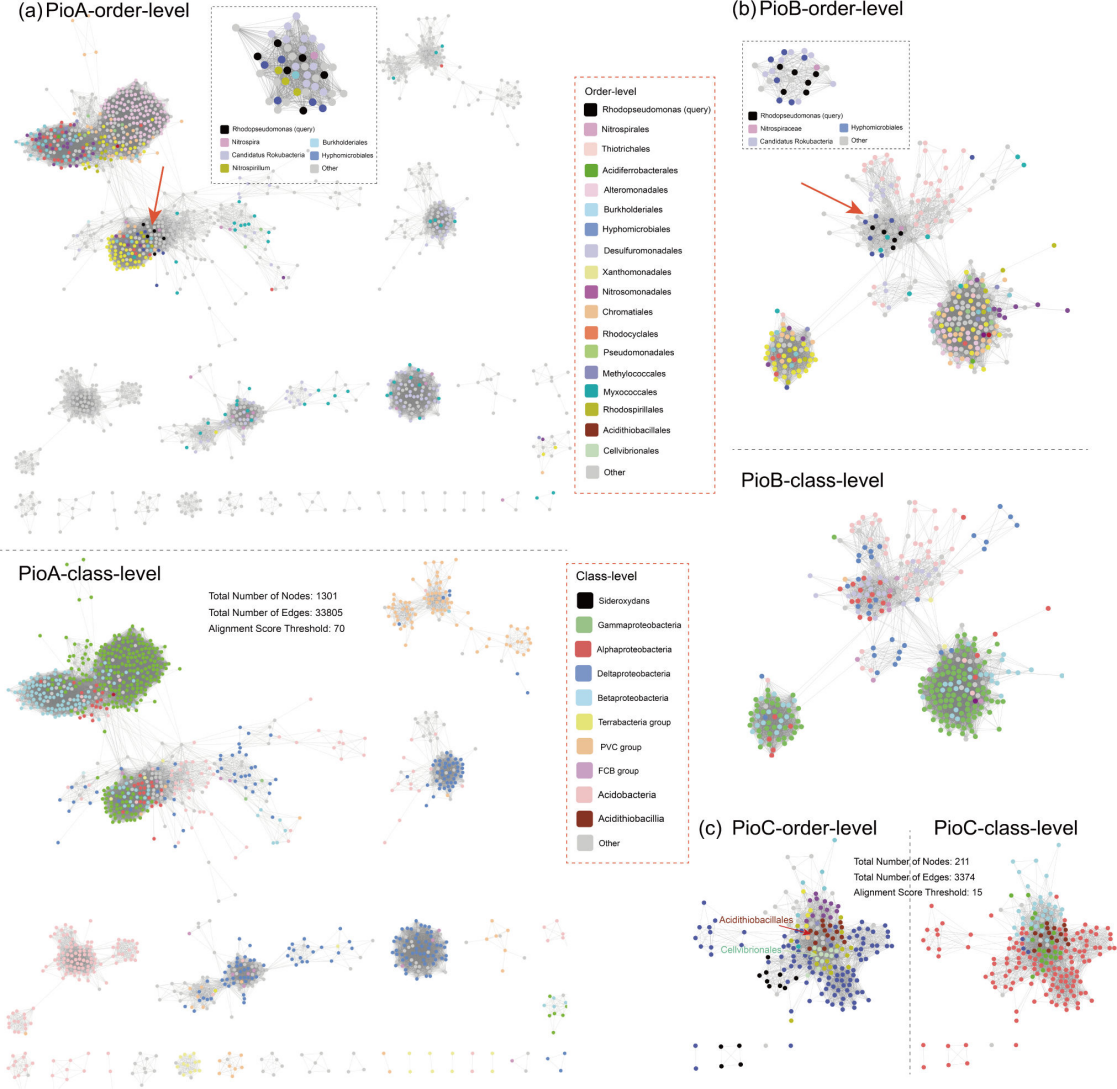
Protein SSNs of representative proteins encoded by the Fe(II) oxidation *pioABC* operon for the visualization of the distribution and evolution diagram of these proteins and their putative homologs: (a) PioA, (b) PioB, and (c) PioC. The sub-SSN showing the first neighbors of the query sequence is marked with a black dotted rectangle frame.

*Rhodopseudomonas* species conduct phototrophic Fe(II) oxidation and iron stress response through proteins encoded by the *pioABC* operon (19 – 21, 54). The *pioABC* operon encodes three proteins: a decaheme cytochrome *c* protein (PioA), an outer membrane porin-like protein (PioB), and a HiPIP protein (PioC). The cytochrome *c* PioA transfer the electrons from Fe(II) to the periplasmic HiPIP PioC, crossing through the outer membrane PioB. Homologs of PioA, PioB, and PioC are widely found in lineages belonging to phyla *Proteobacteria* and *Acidobacteria*. Candidatus *Rokubacteria* (Bacteria *incertae sedis*) are frequently present in the first neighbors of both *Rhodopseudomonas’* PioA and PioB, sharing ~43.0% sequence identity. This suggests a possible novel iron oxidizer (Candidatus *Rokubacteria*) and the occurrence of gene exchange (Fig. 5).

In another neutrophilic bacteria, *Sideroxydans lithotrophicus,* Fe(II) oxidation is achieved through a Fe(II)-oxidizing decaheme cytochrome *c* protein called MtoA, nested within the outer membrane protein MtoB. Fe(II) is oxidized at the bacterial surface, and electrons are transferred to the quinone pool via the tetraheme cytochrome *c* protein CymA and a short cytochrome *c* protein MtoD (7, 22). The SSN diagrams show that the homologs of these components are common in the phylum *Proteobacteria*, with lineages from classes *Gammaproteobacteria* and *Betaproteobacteria* often dominating the hub-like cluster (see Fig. S11 at https://doi.org/10.6084/m9.figshare.20079053.v4). Interestingly, homologs of PioB (Uniprot: A0A0S8L100) and CymA (Uniprot: A0A0S8BIT0/A0A0S8GBH4) belonging to *Acidithiobacillales*, the aforementioned model iron-oxidizing acidophile, are also detected for the first time as far as we know.

The predicted models of *Rhodopseudomonas’* PioAB and *Sideroxydans’* MtoAB show the best PDB hits to MtrAB from the iron-reducing bacterium *Shewanella baltica* (55) (PDB 6R2Q). This suggests that they may traverse the bacterial outer membrane as an electrical bridge, connecting the extracellular and intracellular electron transfer networks. The PioA and MtoA polypeptides, like MtrA, exhibit very few regular secondary structures, consisting mainly of flexible loops (60.6% and 64.3%, respectively), with 37.8%/34.2% helices and 1.6%/1.5% sheets. Additionally, PioA proteins from *Rhodopseudomonas* species have a unique extended N-terminal irregular helix-loop region (residue 1–270) and no heme-binding motif (see Fig. S12 at https://doi.org/10.6084/m9.figshare.20079053.v4). This region is rarely found in PioA homologs from lineages outside of *Rhodopseudomonas*. It is proposed that the periplasmic apo-PioA undergoes a proteolytic process for better heme incorporation. On the other hand, the non-heme holo-PioA_C_ is necessary for the proper incorporation of PioB into the outer membrane. Any mutation in either the signal peptide residue or the PioA_C_ region will result in the loss of phototrophic Fe(II) oxidation ability (20).

Ten conserved CxxCH motifs and distal histidines that encode well-aligned and connected heme coordinating positions are identified in proteins PioA/MtoA/MtrA. These motifs form an electron transfer bridge (see Fig. S12 at https://doi.org/10.6084/m9.figshare.20079053.v4) and suggest evolutionary connections. PioB and MtoB are proposed to associate with PioA/MtoA, forming a porin:cytochrome complex similar to the MtrAB complex (PDB 6R2Q) (55). The predicted 3D models of PioB and MtoB components exhibit porin-like topology, with 26 antiparallel β strands dominated by hydrophobic residues that confer solubility of the Pio/Mto complex in the lipidic outer membrane. PioB and MtoB are proposed to orient and entrap PioA/MtoA in a manner similar to MtrB’s interaction with MtrA, ensuring that the heme chain is perpendicular to the membrane for optimized electron transfer routes (Fig. 1c and d).

CymA is a novel group of inner membrane tetraheme *c*-type cytochrome (c-Cyt) that belongs to the NapC/NrfH quinol dehydrogenase family (23). CymA oxidizes the (mena)quinol in the inner membrane and transfers the released electrons to PioA/MtoA/MtrA directly or indirectly through other periplasmic proteins. The predicted 3D model of *Sideroxydans lithotrophicus* CymA (SlCymA) comprises 60.4% helix and 39.6% coil and contains four heme-binding motifs (CxxCH). The SlCymA model shows the closest structural resemblance (RMSD 3.3 Å, sequence identity 21.0%) to *Desulfovibrio vulgaris* cytochrome *c* nitrite reductase NrfH (56) (PDB 2VR0). The first N-terminal helix of SlCymA (from Ala20 to Phe42), rich with hydrophobic residues, is predicted to be a transmembrane helix by both SOSUI (57) and TMHMM (58) (see Fig. S13 at https://doi.org/10.6084/m9.figshare.20079053.v4). This helix is suggested to be embedded perpendicularly in the membrane for attachment, similar to NrfH (56). NrfH is characterized by the presence of Met49 residue in the heme I binding motif (CxxCHxM) for heme I coordination, with a negatively charged residue (Asp89) acting as the heme-iron distal ligand (56). Both Met49 and Asp89 are essential for menaquinol oxidation, and their counterparts (Met61 and Glu107) are also found in the 3D model of SlCymA at similar positions (see Fig. S13 at https://doi.org/10.6084/m9.figshare.20079053.v4). Identical features of SlCymA are also found in CymA of *Acidithiobacillales bacterium* SM23_46 (Uniprot: A0A0S8GBH4, see Fig. S13 at https://doi.org/10.6084/m9.figshare.20079053.v4). Additionally, we observed that Tyr192 and Trp134, Asp93 and Arg143 in SlCymA (matching to Tyr198 and Trp140, Asp98 and Arg149 in *Acidithiobacillales* CymA-SM23_46), provide additional cross-strand pi-pi/cation interaction. Moreover, Glu60 and Lys111 of SlCymA (matching to Glu65 and Lys117 of CymA-SM23_46) provide additional cross-strand hydrogen bond. These interactions may contribute to the stabilization of CymA, but they are absent in the NrfH homologs (see Fig. S13d at https://doi.org/10.6084/m9.figshare.20079053.v4, highlighted with yellow rectangles).

### The *foxEYZ* operon

The *foxEYZ* operon is required for phototrophic Fe(II) oxidation in several members of *Alphaproteobacteria* (e.g.*, Rhodobacter, Rhodoferax*). It encodes a bihemic cytochrome *c* protein (FoxE) that extracts electrons from Fe(II), a periplasmic protein (FoxY) containing the redox cofactor pyrroloquinoline quinone and an inner membrane transport protein (FoxZ) (26). We find that *foxEY*-like operon is also present in lineages distant to *Rhodobacter*, such as *Rhodocyclales* and *Burkholderiales* (*Betaproteobacteria*), and *Rhodospirillaceae* (*Rhodospirillales*). Homologs from these taxa form a dense cluster (with high pairwise similarity, ~43.2%) in the SSN diagrams, which indicates the occurrence of cross-class/order HGT events. However, homolog of *foxZ* is usually missing in these taxa (see Fig. S14 and S15 at https://doi.org/10.6084/m9.figshare.20079053.v4).

**Fig 6:**
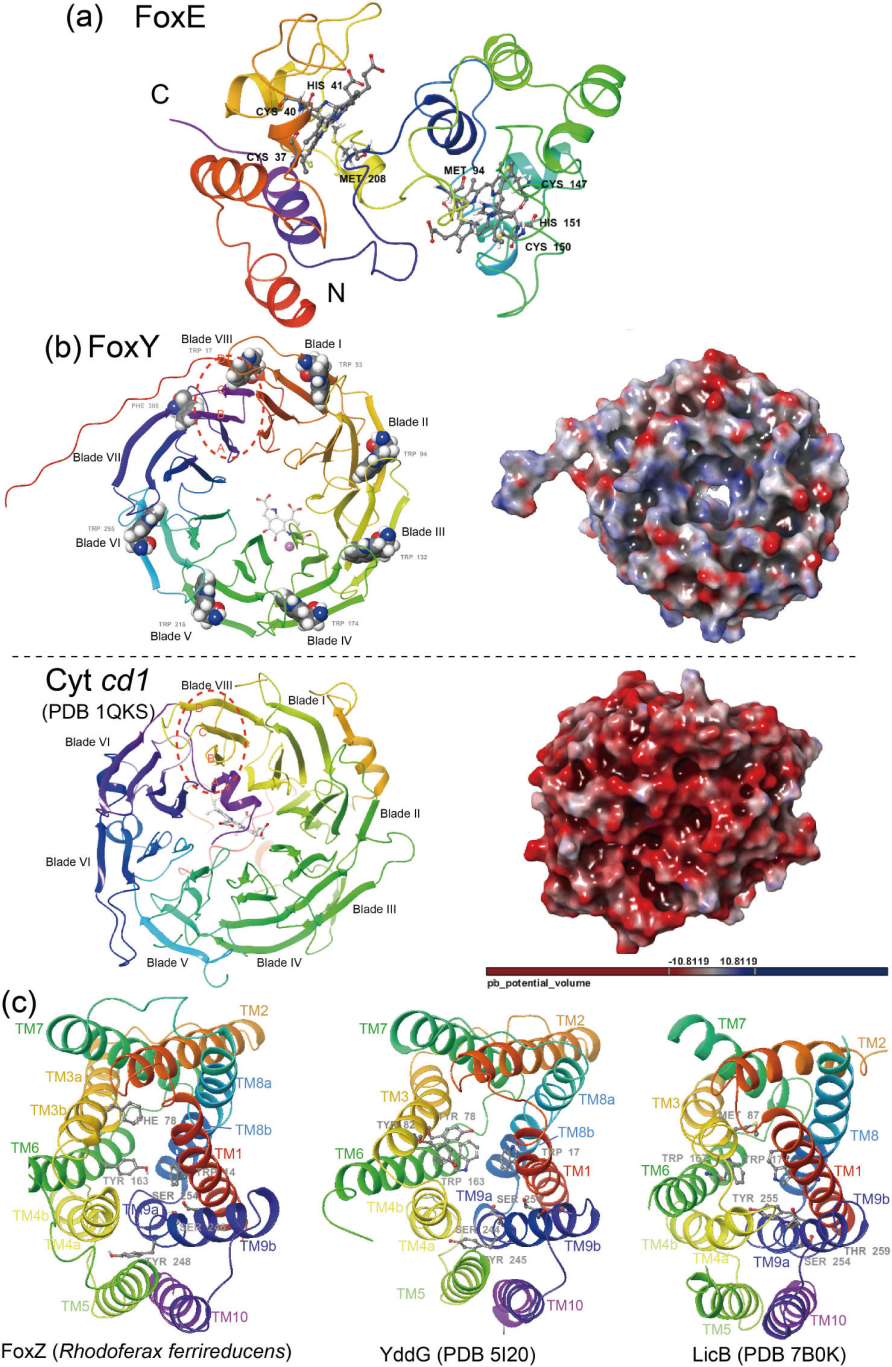
Predicted structures of proteins encoded by operon *foxEYZ* from *Rhodoferax ferrooxidans*. (a) The bihemic cytochrome *c* protein FoxE; (b) FoxY, a propeller-like super-barrel with the girdle of tryptophan residues in the “W” motifs involved in docking the β-sheets together (top) in comparison with cytochrome *cd_1_* nitrite reductase (PDB 1QKS, bottom). The electrostatic potential surfaces are calculated with adaptive Poisson-Boltzmannsolver (APBS); (c) FoxZ with the drug/metabolite transporter (DMT) superfamily fold and comparisons among FoxZ, *Starkeya novella* YddG (PDB 5I20) and *Streptococcus pneumoniae* LicB (PDB 7B0K). The overall fold of the polypeptide chain is ramp-colored from red (N-terminal) to purple (C-terminal).

The 3D model of *Rhodoferax ferrireducens* FoxE, in resemblance to *Rhodobacter ferrooxidans* FoxE (PDB 5MAB, with 65% sequence identity and RMSD 1.8 Å) is predicted to have a di-heme topology dominated by alpha helices (Fig. 6a; see Fig. S16 at https://doi.org/10.6084/m9.figshare.20079053.v4). The *R. ferrireducens* FoxE polypeptide chain starts with a short protruding helix from N-terminus, followed by a hydrophobic structural core that contains two hemes covalently coordinated by conserved residues Cys37-Cys40-His41-Met208 and Met94-Cys147-Cys150-His151, respectively, with methionines occupying the distal axial positions of heme I and II. Besides, heme I and II of *R. ferrireducens* FoxE exhibit distinct orders in the attachment of the heme-binding motif CxxCH and the axial methionine ligand: methionine is preceding the CxxCH motif of heme II, but in heme I, the CxxCH motif is way ahead of the turn-back C-terminus Met208. In addition, a disulfide bridge between cysteines Cys58 and Cys236 (refer to Cys80 and Cys258 of PDB 5MAB) seems to supply an auxiliary covalent bond to sustain the conformation of the turn-back C-terminus (Fig. 6a).

The predicted 3D structure of FoxY is a compact propeller-like super-barrel with eight topologically identical propeller blades positioned radially around the protein center in pseudo-eightfold symmetry (Fig. 6b). Each β-propeller blade composes four antiparallel β strands (A–D), with the short A strand closest to the pseudo-eightfold axis and the D strand on the subunit surface. A typical tryptophan-docking (W) motif for structure stabilization is located on each strand D, except in W7 where a Phe308 replaces the common tryptophan residue (Fig. 6b and c). These aromatic residues putatively contribute to the electron transfer channel, as predicted by eMap (59) (see Fig. S17 at https://doi.org/10.6084/m9.figshare.20079053.v4). The topological features mentioned above also exist in peripheral membrane proteins, including cytochrome *cd_1_* nitrite reductase (60) (PDB 1QKS, with RMSD 2.4 Å), methanol dehydrogenase (61) (PDB 1H4I, with RMSD 3.0 Å), and BamB (62) (PDB 4HDJ, with RMSD 2.8 Å against FoxY) (see Fig. S18 at https://doi.org/10.6084/m9.figshare.20079053.v4). The similarity in structure between the iron respiratory component FoxY and the redox cytochrome *cd_1_* protein is intriguing in light of their putative evolutionary connection. Upon detailed comparison, it is observed that FoxY lacks the extended N-terminus loop region that harbors the heme-binding motif (CxxCH) seen in cytochrome *cd_1_* (PDB 1QKS), and the other loops of FoxY are generally much shorter than those of cytochrome *cd_1_*. Furthermore, the overall surface change of FoxY appears to be less negative compared to cytochrome *cd_1_* (Fig. 6b). Besides, the strands A–C of FoxY blade W8 (closure of the overall ring structure) derive from the C-terminus, while the D strand comes from the N-terminus. However, cytochrome *cd_1_* has a distinct blade W8 constitution that is made up of strands B–D coming from the N-terminus, plus an A strand from the C-terminus (Fig. 6b, highlighted with red dotted circles).

The overall predicted structure of FoxZ exhibits the basket-like DMT superfamily fold (Fig. 6c). A structural homolog search using the 3D model of FoxZ revealed significant structural resemblance to the distant DMT superfamily inner membrane protein (e.g., *Starkeya novella* YddG (63) (PDB 5I20) and *Streptococcus pneumoniae* LicB (64) (PDB 7B0K), which are involved in the transport of cationic compounds. These proteins superimpose with an RMSD of about 2.2  Å and have a sequence identity of approximately 21.0% (see Fig. S19 at https://doi.org/10.6084/m9.figshare.20079053.v4). Similar to other DMT family proteins, the 3D model of FoxZ consists of 10 transmembrane α-helices (TM), with both N- and C-termini situated on the cytoplasmic side and adopting an outward-open conformation. The overall topology of FoxZ comprises two inverted and repeated halves, with the N-terminal TM1 to TM5 domains and the C-terminal TM6 to TM10 parts arranged in twofold antiparallel pseudo-symmetry surrounding a central cavity. TM5 and TM10 in alliance with TM4 and TM9 form a bundle that contributes to the central cavity. It is observed that TM3, TM4, TM8, and TM9 of FoxZ are interrupted by short loops, forming short helical segments (e.g., TM4a, TM4b). Note that TM3 of YddG (PDB 5I20) and TM8 of LicB (PDB 7B0K) are consecutive. Besides, various conserved and essential aromatic residues previously reported to form an “aromatic box” surrounding the central cavity and participate in ligand coordination are also identified in similar positions of the FoxZ structure. These residues include Trp14 (TM1), Phe78 (TM3), and Trp163 (TM6) of FoxZ in correspondence to Trp17, Tyr78, and Trp163 of YddG (PDB 5I20) and Trp17, Tyr78, and Trp163 of LicB (PDB 7B0K). Moreover, conserved hydrophilic residues in the central cavity like Ser246 (TM9) and Ser254 (TM9) of FoxZ (in reference to Ser244 and Ser251 of YddG) are also discovered, which may supply binding sites for the hydrophilic groups of ligands (64) (Fig. 6c).

### Iron oxidase in *Zetaproteobacteria*

The predicted Fe(II) oxidation pathway in marine *Zetaproteobacteria* strain *Mariprofundus ferrooxydans* PV-1 involved a Cyc2-like iron oxidase (GenBank: AKN78226) and probably the Mob protein (SPV1_03948, also named as ActB), a Fe–S molybdopterin oxidoreductase on the inner membrane that is significantly expressed in PV-1 cells under Fe(II) oxidizing conditions and may play a significant role in the electron transport during Fe(II) oxidation (8, 28). An SSN analysis of the Mob protein revealed that the closest relatives of *M. ferrooxydans* are primarily lineages from *Rhodocyclales* (*Betaproteobacteria*) and *Rhodospirillales* (*Alphaproteobacteria*) (see Fig. S20 at https://doi.org/10.6084/m9.figshare.20079053.v4). Besides, the genome contexts of the Mob protein are unconserved (see Fig. S21 at https://doi.org/10.6084/m9.figshare.20079053.v4), indicating putative HGT events.

The predicted 3D model of *Mariprofundus ferrooxydan* Mob protein demonstrates significant structural homology to the ArrA subunit of arsenate respiratory reductase from *Shewanella* sp. ANA-3 (PDB 6CZ7) with an RMSD of 2.6 Å and a sequence identity of 22.1%. The Mob protein exhibits the typical four-domain topology commonly observed in Mo-bisPGD enzymes (65, 66) (see Fig. S22 at https://doi.org/10.6084/m9.figshare.20079053.v4). In Mob, the (4Fe–4S) cluster is coordinated by Cys74, Cys77, Cys81, and Cys109 (analogous to Cys61, Cys64, Cys68, and Cys96 in ArrA). Additionally, Mob contains residues that are presumed to hydrogen-bond with the substrate, including Thr166, Phe189, Tyr208 (corresponding to Tyr166, His189, and Tyr210 in ArrA), and Thr191 and His198 (matching Ser190 and Lys198 in ArrA), which likely function at the oxidized Mo(VI)O and reduced Mo(V)OH forms of the protein, respectively (65, 66) (see Fig. S22 at https://doi.org/10.6084/m9.figshare.20079053.v4).

### Models of archaeal Fe(II) oxidization

In the proposed model for *Metallosphaera* spp., a group of acidophilic and thermophilic archaea, the *fox* operon-related Fe(II) oxidation process involves the extraction of electrons from Fe(II) by cytochromes *b* FoxCD, which are then transported through a multi-copper oxidase (Mco) to the heme copper oxidase FoxAB for O_2_ reduction (29 – 31). The SSN analysis reveals that the closest non-*Sulfolobales* sequence homologs of *Metallosphaera*’s FoxA come from *Gammaproteobacteria* and *Cyanobacteria* (see Fig. S23 at https://doi.org/10.6084/m9.figshare.20079053.v4). Phylogenetic analysis suggests that the FoxBCD may exist in early *Sulfolobaceae*, exhibiting close homology to the Fox proteins from archaeal phylum Candidatus *Marsarchaota* (see Fig. S23 through S26 at https://doi.org/10.6084/m9.figshare.20079053.v4). FoxA aligns best with subunit I of bovine *aa_3_*-type cytochrome *c* oxidase (PDB 5IY5) with an RMSD of 2.3 Å and 19% sequence identity, as determined by DALI (46) (see Fig. S27 at https://doi.org/10.6084/m9.figshare.20079053.v4). Similarly, the predicted 3D model of FoxB shows similarity to quinol oxidase subunit CyoA of *Escherichia coli* (PDB 1CYX) with RMSD 2.3 Å and 28% sequence identity (see Fig. S28 at https://doi.org/10.6084/m9.figshare.20079053.v4). On the other hand, FoxC exhibits the highest resemblance to the ethylbenzene dehydrogenase γ-subunit (PDB 2IVF) with RMSD 3.5 Å and 15% sequence identity, which exhibits a crescent-like structure where a noncovalently bound heme *b* is tightly packed (see Fig. S29 at https://doi.org/10.6084/m9.figshare.20079053.v4) (67). COFACTOR (68) predicts the presence of a binding pocket for the [Fe(4)S(4)] cluster in FoxC, formed by the residues Q45, I46, N47, and W57. Both FoxB and FoxC display an overall negatively charged electrostatic potential surface. Lastly, the predicted 3D model of FoxD appears to be homologous to chain A of nitric oxide reductase of *Geobacillus stearothermophilus* (PDB 3AYF), with a TM-score of 0.51 and an RMSD of 5.6 Å, although the sequence identity is relatively low (6.7%). Additionally, COFACTOR (68) assigns FoxD the molecular function of cytochrome *c* oxidase activity (GO:0004129, score 0.51) and the biological process of hydrogen ion transmembrane transport (GO:1902600, score 0.48). A heme A-binding pocket is predicted to exist in FoxD, formed by residues including R3, F12, Y16, E23, R80, and S306. Lastly, the predicted 3D model of Mco shares structural similarity with rusticyanin (PDB 1CUR) with an RMSD of 2.6 Å and a sequence identity of 27.5%.

The bioenergetic pathways of the acidophilic archaeon *Ferroplasma* spp. appear to be relatively simple. These pathways consist of a fusion protein of heme/copper-type oxidase subunits I and III , as well as a stand-alone subunit II. Additionally, there is a copper protein known as sulfocyanin that associates with the oxidase subunit II (32). Sulfocyanin is proposed to serve as the primary electron acceptor from Fe(II) and directly interacts with the substrate at the cell surface (Fig. 1h). The predicted 3D model of sulfocyanin displays an RMSD of 2.9 Å and a weak sequence identity (14%) to rusticyanin (PDB 1CUR). The fusion protein of putative oxidase subunits I and III bears a close resemblance to *Mycobacterium tuberculosis* cytochrome *c* oxidase subunit 1 (PDB 7E1V) with RMSD 1.6 Å and 13% sequence identity. Conversely, subunit II shares homology with the quinol oxidase subunit CyoA of *Escherichia coli* (PDB 1CYX) with RMSD 2.2 Å and 28% sequence identity.

### Distribution of Fe(II) oxidation proteins

**Fig 7:**
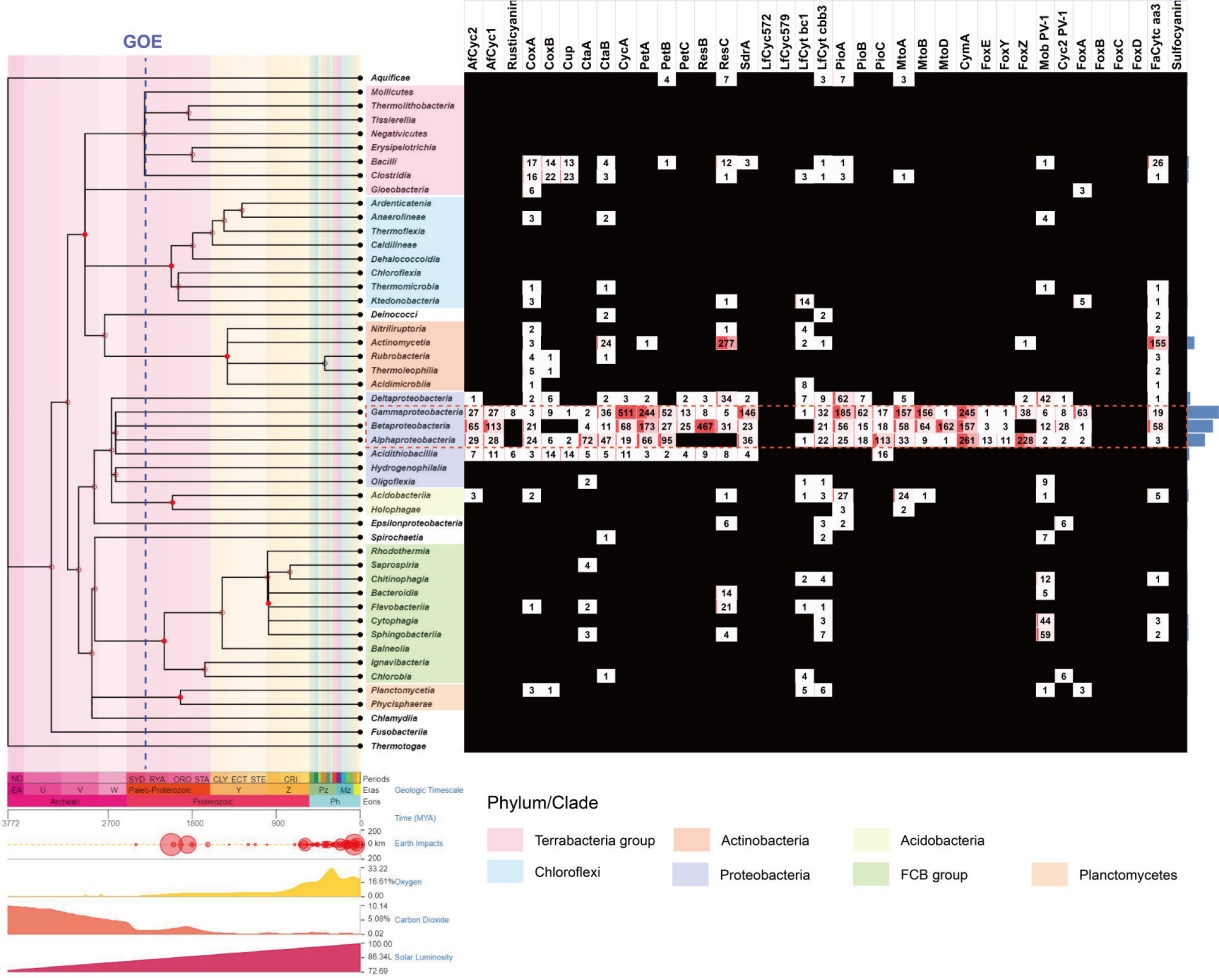
Distribution of Fe(II) oxidation proteins at phylum level assessed by querying the non-reductant UniRef_90 database (*E*-value 1e–20). Data of asteroid impacts, solar luminosity, and fluctuations of atmospheric oxygen and carbon dioxide amount provided by Timetree (http://www.timetree.org) were displayed synchronously with divergence times in the form of time panels. The estimated occurrence time of the GOE event (~2,400 MYA) was marked with a blue dotted line.

The SSN diagrams above illustrate that homologs of characterized iron oxidation proteins are often found across a wide range of taxa, extending beyond model organisms. It is possible that these organisms also possess similar Fe(II) oxidation functions. However, we have limited knowledge about the extent to which these Fe(II) oxidation proteins diversify. To gain more insight into the overall evolution of microbial iron oxidation pathways, we performed an assessment and summary of the distribution of homologs of the previously mentioned Fe(II) oxidation proteins. We queried the non-reductant UniRef_90 database (69) (*E*-value 1e–20). Our results revealed a widely extended taxon profile, in which the components of the *rus, pet, pio, mto,* and *foxEYZ* operons are sparsely distributed overall but highly enriched in classes *Gammaproteobacteria* (with a total of 2,084 hits), *Betaproteobacteria* (with a total of 1,684 hits), and *Alphaproteobacteria* (with a total of 1,168 hits) (Fig. 7, highlighted with a red dotted rectangle). According to Timetree (70), these taxa are predicted to have originated around 2,621 million years ago (MYA), before the Great Oxidation Event (GOE, ~2,400 MYA) (Fig. 7, left). Surprisingly, homologs of the Mob protein are particularly enriched in the classes *Sphingobacteriia* (59 hits) and *Cytophagia* (44 hits), whereas considerable PioA/MtoA homologs are also identified in classes *Acidobacteriia* (27 hits) and the deep-branching bacterial extremophile *Aquificae* (seven hits) (Fig. 7).

Specifically, the following genera have been found to simultaneously possess nearly all necessary components of *pet, pio, mto,* and *foxEYZ* operons: *Sedimenticola* (*Gammaproteobacteria*)*, Thauera, Rhodoferax* (*Betaproteobacteria*), *Magnetospirillum*, and *Rhodovulum* (*Alphaproteobacteria*) (refer to Table S1 at https://figshare.com/articles/dataset/SI_docx/19939805/2, marked with red color). Additionally, rich with components of *pet* and *pio/mto* operons are *Gammaproteobacteria* genera such as *Halomonas, Vibrio*, and *Photobacterium; Betaproteobacteria* genera such as *Noviherbaspirillum, Rhizobacter*, and *Rubrivivax;* and finally, *Alphaproteobacteria* genera *Bradyrhizobium* and *Nitrospirillum* (see Table S1 at https://figshare.com/articles/dataset/SI_docx/19939805/2, marked with yellow color). Furthermore, the genera *Kordiimonas, Azospirillum, Telmatospirillum* (*Alphaproteobacteria*), and *Marinimicrobium* (*Gammaproteobacteria*) possess homologs of *pio/mto* operons (see Table S1 at https://figshare.com/articles/dataset/SI_docx/19939805/2, marked with green color) while genera *Roseospira, Magnetospira, Magnetovibrio, Rhodobacter* (*Alphaproteobacteria*), and *Leptothrix* (*Betaproteobacteria*) solely contain homologs of *foxEYZ* operon components (see Table S1 at https://figshare.com/articles/dataset/SI_docx/19939805/2, marked with blue color). Intriguingly, genus *Pseudomonas* (*Gammaproteobacteria*) is also abundant with homologs of CycA (78 hits), CymA (22 hits), and Cyc1 (17 hits) (see Table S1 at https://figshare.com/articles/dataset/SI_docx/19939805/2, marked with a bold black rectangle).

## DISCUSSION

In this study, we present a comprehensive analysis of the distribution and evolution of experimentally characterized microbial ferrous iron oxidation pathways. Our approach includes SSN analysis, phylogenetic analysis, genomic analysis, and structural comparisons. Through these methods, we have discovered a diverse range of taxa that possess homologs to known iron oxidation proteins. This finding suggests the potential of our method for the identification and characterization of novel Fe(II) oxidizers.

To summarize, our study yields two main findings regarding the evolution of microbial Fe(II) oxidation pathways:

(a) Quite a number of acknowledged proteins involved in diverse Fe(II) oxidation pathways are structural homologs, exhibiting similar three-dimensional structures and functional characteristics to proteins involved in other Fe(II) oxidation pathways (Table 2).

**TABLE 2:** Structure commonality in iron oxidation proteins from various taxa

Protein	Taxa	Function	Topological commonality	TM-align score	Sequence identity
Cyc2	*Acidithiobacillus*	Outer membrane porin-cytochrome protein	β-barrel porin-like topology	0.50–0.74	6%–16%
Cyc2-PV1	*Mariprofundus*	Cyc2-like iron oxidase			
Cyc572	*Leptospirillum*	Outer membrane cytochrome *c*			
PioB	*Rhodopseudomonas*	Outer membrane protein			
MtoB	*Sideroxydans*	Outer membrane protein			
PioC	*Rhodopseudomonas*	High-potential iron-sulfur protein	Conserved cysteine residues ligating the [Fe(4)S(4)] cluster	0.8	15%
Iro	*Acidithiobacillus*	Iron oxidase, high-potential iron-sulfur protein			
Rusticyanin	*Acidithiobacillus*	Electron transmitter	Common core topology consisting of a β-barrel or sandwich core and two mixed β-sheets	0.47–0.64	11%–14%
Mco	*Metallosphaera*	Multi-copper oxidase			
Sulfocyanin	*Ferroplasma*	Primary electron acceptor from Fe(II)			

AI-driven accurate protein prediction, such as RoseTTAFold (39), was selected by the journal *Science* as the top 1 breakthrough (71). With this approach, we have observed a striking pattern among Fe(II) oxidizers, which utilize a porin protein as the initial component of the iron respiratory chain to interact with the external environment/substrate. This may be attributed to convergent evolution. Cyc2 from *At. ferrooxidans/M. ferrooxydans,* Cyc572 from *Leptospirillum* spp., and PioB/MtoB/MtrB from neutrophilic bacteria all display a β-barrel porin-like topology, with pairwise TM-align score ranging between 0.50 and 0.74 (TM-score over 0.5 is considered as having similar fold), despite the low sequence identities observed (6%–16%).

Porins are integral membrane proteins primarily found in the outer membranes of Gram-negative bacteria and mitochondria. They typically assume a β-barrel topology (72). In the context of Fe(II) oxidation, porin proteins serve as the initial component of the iron respiratory chain (73). They function by facilitating the transport of Fe(II) ions across the bacterial outer membrane and into the periplasmic space. The interaction between Fe(II) oxidizers and their external environment/substrate is critical for their survival and energy production. By utilizing the porin protein, the microorganisms establish a direct connection between their cellular machinery and the external iron source, facilitating the extracellular electron transfer from the Fe(II) substrate to the subsequent components of the iron respiratory chain. Cyc2, MtoAB, and PioAB are all involved in iron oxidation in neutrophilic bacteria while MtoAB and PioAB have not been reported in acidophilic bacteria. These proteins play roles in extracellular electron transfer and iron mineralization processes (73). The functional similarity also suggests a potential evolutionary relationship among these proteins.

The β-barrel cytochrome-porin fusion proteins, such as Cyc2 and Cyc572, appear to represent the more primitive evolutionary product of outer membrane porin proteins that have occasionally incorporated a heme-binding motif through gene fusion events. These fusion proteins are efficient in acidophilic iron oxidizers, such as *Acidithiobacillus*, and have gradually become dominant in nature (27). We propose that this cytochrome-porin iron-oxidizing fusion protein has later evolved into the complex form (MtoAB/PioAB) observed in neutrophilic iron oxidizer. This proposal is supported by several factors. Firstly, the cumulative number of protein folds generally increases over time (74). We observed that the number of β strands and radius of porin increased from approximately 17 β strands of Cyc2 to 26 β strands of PioB/MtoB/MtrB and 38.2 Å of Cyc2 to 50.4 Å of PioB/MtoB/MtrB, respectively. This is consistent with the proposal that acidophilic iron oxidizer emerges prior to neutrophilic iron oxidizer inhabiting fresh and marine waters (75). Secondly, the enlargement of the porin size of PioB/MtoB/MtrB may be necessary to accommodate the trapped, large, and elongated cytochrome/filament that contains abundant closely stacked hemes, such as PioA/MtoA/MtrA, that have evolved from c552-family cytochromes (76). The microbial Fe(II) oxidation at neutral pH is typically mediated by extracellular electron transfer (EET) mechanisms (35, 76, 77), which is guaranteed by the above configuration. EET involves the transfer of electrons from Fe(II) to extracellular electron acceptors, such as cytochromes, which are often associated with the outer membrane of microorganisms. This extracellular electron transfer process may help the neutrophilic iron oxidizers to counteract the problem of the insolubility of Fe(III) at neutral pH, where ferric hydroxide precipitates can clog around or inside the cell (35).

Resemblance in the folding structure is also observed between high-potential [4Fe-4S] iron-sulfur proteins (HiPIP), such as Iro from *At. ferrooxidans* and PioC from *Rh. palustris* (pairwise TM-align score 0.78), despite their low sequence identity of 15%. The conserved cysteine residues, Cys24, Cys27, Cys36, and Cys49 (in the order Iro), are crucial for ligating the [Fe(4)S(4)] cluster. Additionally, the aromatic residues Tyr14, Phe30, and Tyr48 (replaced by Trp86 in PioC), which play a significant role in protein stabilization and electron transfer, have been identified (78). The *pioC* gene is the final gene found in the *pio* operon and may have been acquired through HGT) and fused with *pioAB* (79). This hypothesis is supported by the presence of a homologous *pioAB* operon in *S. lithotrophicus*, known as the *mtoAB* cluster. However, the *mtoAB* cluster lacks a gene encoding HiPIP, which is instead replaced by the *cymA* gene encoding a membrane-anchored cytochrome *c* (Fig. S3B) (7). Additionally, the *pioC* in-frame deletion mutant showed only a partial defect in Fe(II) oxidation, leading to the proposal that PioC could be replaced by other unknown small soluble electron carriers (19 – 21). The presence of HiPIP with redox potentials ranging from +0.05 to +0.5 V may have allowed for adaptation to the elevated oceanic redox potential resulting from oxygenation events (80).

Another example of resemblance in folding structure is observed among rusticyanin from *At. ferrooxidans,* sulfocyanin from *F. acidarmanus,* and Mco from *Metallosphaera* spp. These proteins demonstrate a common core topology, consisting of a β-barrel/sandwich core and two mixed β-sheets. The pairwise TM-align scores for these proteins range from 0.47 to 0.64. Despite a low sequence identity of 11%–14%, these proteins conserve key residues involved in copper ion binding. Specifically, the order of these residues in rusticyanin is His85/His143/Cys138/Met148, while sulfocyanin has Thr114 instead of Ser86. These residues are crucial for maintaining acid stability and modulating redox potential by facilitating hydrogen bond interactions (81, 82).

The significant fold homology but low sequence identity observed in multiple iron oxidation proteins is similar to the characteristics observed in heat shock proteins and G protein-coupled receptors (83 – 85). There are several possible scenarios that may account for this similarity:

1. Ancient origin hypothesis: The iron oxidation gene cassette could have evolved in the last common ancestor. Although there is limited direct evidence, this hypothesis is supported by shared characteristics and conserved genes involved in iron oxidation among diverse microbial groups. In their review article titled “Geomicrobiology of Iron,” Kappler et al. (86) provide an overview of iron oxidation in various microbial lineages and discuss the potential ancient origin of these pathways.

2. HGT and later divergence: An alternative hypothesis suggests that the ability to oxidize ferrous iron arose independently in different lineages through horizontal gene transfer events. This scenario proposes that genes associated with iron oxidation were acquired through HGT, leading to the establishment of iron oxidation pathways in various microbial groups after their divergence. In the study conducted by He et al. (35), a thorough analysis of neutrophilic iron(II) oxidizer genomes was carried out to investigate the potential acquisition of genes related to iron oxidation through HGT. It was consistently found that both the iron sulfur (Fe/S) protein and cytochrome *c* domains appeared in Bacteria and were later horizontally transferred to Archaea (via HGT) and Eukarya (via endosymbiosis) (87 – 89). Moreover, the giant mobile genetic elements known as “Borgs” frequently contain a variety of multi-copper oxidases, cupredoxins, and multi-heme cytochromes (90).

3. Interplay of vertical and horizontal evolution: It is possible that both vertical inheritance and HGT have contributed to the diversity of iron oxidation pathways. This theoretical scenario proposes that the last common ancestor possessed a primitive iron oxidation pathway which underwent subsequent modification and diversification through both vertical evolution and the acquisition of novel genes via HGT. This idea is supported by a study conducted by Barco et al. (28), which investigates the proteomic profile of an obligate iron-oxidizing chemolithoautotroph and highlights the interplay between vertical and horizontal evolution in shaping iron oxidation systems.

(b) Lineages belonging to classes *Gammaproteobacteria* and *Betaproteobacteria* are frequently found at the central positions of SSNs of Fe(II) oxidation protein, from which homologs of other taxa are derived.

The analyses of SSNs divide the protein family into clusters based on their sequence similarities, providing a more intuitive and quick visualization for the analysis of the taxonomic distribution and functional space of the target protein family (91). The hub cluster in an SSN may represent a more primitive form of proteins from which other clades (homologs) derived (92). Based on this assumption and our findings, it is proposed that many known microbial Fe(II) oxidation pathways may have originated within classes *Gammaproteobacteria* and *Betaproteobacteria*.

There are two additional pieces of evidence to complete the picture: (i) a significantly large number of homologs of the known Fe(II)-oxidizing related proteins are identified in classes *Gammaproteobacteria* (2,084 hits) and *Betaproteobacteria* (1,684 hits) compared to other taxa, and among these hits, several Fe(II) oxidation proteins (e.g., PioAB/MtoAB, and CymA) appear to be more widely distributed than the others; (ii) classes *Gammaproteobacteria* and *Betaproteobacteria* are predicted by Timetree (70) to have diversified from their last common ancestor (~2,621 MYA) prior to the GOE (~2,400 MYA, Fig. 7, left), whereas most other taxa that have sparsely distributed Fe(II)-oxidizing components are predicted to originate at a time period after the GOE (Fig. 7). For example, class *Acidobacteriia* that contains PioA/MtoA homologs is suggested to originate at around 2,009.3 MYA, and classes *Sphingobacteriia* and *Cytophagia* that harbor homologs of Mob protein are proposed to be present at about 920.1 MYA (70).

These findings provide support for the hypothesis that most key Fe(II) oxidation proteins originated within *Proteobacteria* branches *Gammaproteobacteria* and *Betaproteobacteria,* which likely emerged in the anoxic oceanic environment where abundant soluble Fe(II) are available due to the continuing weathering of the continental crust along with deep-sea hydrothermal convection prior to the GOE. In addition, these Fe(II) oxidation proteins likely performed reversible anoxygenic Fe(II) oxidation utilizing nitrate/nitrite (as an electron acceptor) rich in the primordial seawater produced through lightning-catalyzed nitrogen conversion (93 – 95). This mechanism is effective as the nitrogen oxide gases produced are continuously removed. Bacterial species that couple the oxidation of Fe(II) to nitrate reduction have been isolated from a wide range of habitats (96, 97). They oxidize both soluble and insoluble Fe(II) (98, 99). Oxidation of Fe(II) may also serve as an important detoxification strategy of toxic reactive nitrogen species in both photosynthetic and nitrate-reducing bacteria (100, 101). The standard membrane-bound supposed pre-last universal common ancestor enzyme, Nar, was reported to serve as the combined Fe(II) oxidase and nitrate reductase (99, 102). Moreover, it is reported that the iron-oxidizing multi-copper oxidase and rusticyanin share evolutionary origin with nitrite reductase (NiR) that could use the accumulating nitrite as an oxidant (103). Nitrite reductase (NrfB) was also found to share conserved heme orientation with the iron-metabolizing Mtr complex (76). Therefore, nitrate-dependent Fe(II) oxidization may represent the most ancient dissimilatory Fe(II) metabolism. Consistently, we found that the acknowledged Fe(II) oxidation proteins such as CymA, FoxY, and FoxD showed significant structural homologies to cytochrome *c* nitrite reductase NrfH (PDB 2VR0), cytochrome *cd_1_* nitrite reductase (PDB 1QKS), and nitric oxide reductase (PDB 3AYF), respectively. Moreover, nitrate-dependent Fe(II) oxidizers are still widespread in both bacterial and archaeal lineages nowadays (73, 98, 99), and several aerobic Fe(II) oxidizers such as *Acidithiobacillus* species (104) and other biometallurgical strains (105) have retained the ability to perform Fe(II) oxidation coupled with sulfate (analog of nitrate) reduction under anaerobic conditions.

From these findings, it can be inferred that these Fe(II) oxidation proteins may have been vertically transmitted for a relatively long period within *Gammaproteobacteria* and *Betaproteobacteria,* allowing for the accumulation of large quantities of homologs and subsequently adapted to the shift of oxygen as the terminal electron acceptor. As previously observed, the GOE has promoted the innovation and diversification of metabolic pathways (106). The vertically transferred process was probably followed by horizontal transfers of these homologs to other distantly related taxa after the GOE, as evidenced by the G + C content deviation, frequent presence of flanking mobile genetic elements in the genomes, and the sporadic and irregular distribution of the Fe(II) oxidation homologs in their genomes (Fig. 2 and 4).

Although these findings offer valuable insights into the topic, investigations on the evolution of microbial iron oxidation pathways remain open. Ongoing studies continue to contribute to our understanding and may offer alternative perspectives.

## MATERIALS AND METHODS

We utilized FeGenie (43) by default parameters to identify candidate genes associated with iron oxidation in the genomes of reported iron oxidizer (see Supplementary Material at https://doi.org/10.6084/m9.figshare.23652387.v1). FeGenie (43) is a computational tool designed for identifying putative iron-related genes from genomic and metagenomic data sets. It utilizes a combination of gene identification and annotation algorithms, along with specific criteria related to iron-associated functions, to predict and retrieve genes potentially involved in iron metabolism and homeostasis. The representative sequences of known Fe(II) oxidation proteins were obtained from the FeGenie results as the initial query sequences for the following analyses. SSNs of the targeted gene families were calculated via the Enzyme Similarity Tool (EFI-EST) (107), with BLAST query *E*-value of 1e–20 against the non-reductant UniRef_90 database (69) for homologous sequences retrieval and *E*-value of 1e–30 for BLAST to calculate similarities between sequences defining edge values. The alignment score threshold for generating the final SSN was selected at the score corresponding to 35% sequence identity which is also indicated in the illustrated SSN diagrams. The UniRef_90 database was built by clustering the UniProt original sequences at cutoffs of 90% sequence identity and 80% overlap with the longest sequence in the cluster (the seed sequence) (69). SSN is visualized with “Organic layout” in Cytoscape v. 3.7.1 (108). Genome Neighborhood Tool (EFI-GNT) (107) was used to analyze the gene context in genomes. The phylogenetic tree based on protein sequences was built using PhyML (109) with the maximum likelihood (ML) method (1,000 bootstrap replicates), followed by visualization with iTOL (110). Sequences were aligned with MUSCLE (111) and trimmed with Gblocks (112) prior to tree construction.

SignalP v.5.0 (113) (https://services.healthtech.dtu.dk/services/SignalP-5.0/) was used for signal peptide prediction. SOSUI (57) (http://harrier.nagahama-i-bio.ac.jp/sosui/) and TMHMM (58) (https://services.healthtech.dtu.dk/services/TMHMM-2.0/) were used for transmembrane region predictions. The local version of RoseTTAFold was configured according to instructions from https://github.com/RosettaCommons/RoseTTAFold (39) on our lab’s computation resource, a Dell PowerEdge R940xa server with four Intel Xeon Platinum 8260 processors (total of 148 cores), 1TB of RAM, installed with Ubuntu 18.04.6 distribution, python 3. Prediction of protein 3D structure of the targeted Fe(II) oxidation protein was conducted with RoseTTAFold (39). The similar structures in the PDB database were found by fold recognition using DALI (46) or COFACTOR (68) with the RoseTTAFold prediction as a query. TM-align program (114) was used for structure comparisons. Visual Molecular Dynamics (VMD) software v.1.9.4 (115) was applied for structural analysis, visualization, and graphics production. Electrostatic potential was calculated with adaptive Poisson-Boltzmann solver (APBS) (VMD APBS Plugin, version 1.3.1). Electron transfer pathway(s) within the targeted protein was calculated with eMap (59) (default parameters), which applied the graph theory to predict electron tunneling through electron transfer active moieties.

### Conclusions

Fe(II) has likely served as an energy substrate for microbial metabolism for billions of years. This study aims to provide a comprehensive understanding of the distribution and evolution of microbial Fe(II) oxidation pathways by integrating SSN analysis and protein structural comparisons. Examining the non-redundant database revealed a surprisingly broad range of taxa, including classes *Gammaproteobacteria* (2,084 hits) and *Betaproteobacteria* (1,684 hits), harboring homologs of the known Fe(II) oxidation proteins. Additionally, evidence of HGT was found in many Fe(II) oxidation proteins. Notably, classes *Gammaproteobacteria* and *Betaproteobacteria* often occupy the hub positions of the protein SSNs from which homologs of other taxa are derived. The RoseTTAFold predictions also provide insights into those structurally unknown Fe(II) oxidation components, such as FoxY and FoxZ. Many proteins involved in diverse Fe(II) oxidation pathways exhibit close structural homology, suggesting convergent evolution. Still, the current Fe(II) oxidation models are far from finished. With the increasing number of pure isolates, genomic/structural data, and biochemical validations available, it is expected that additional Fe(II) oxidation mechanisms and evolutionary details will be uncovered.

## Data Availability

All this study’s seed sequences and RoseTTAFold structural predictions are available to the community via https://doi.org/10.6084/m9.figshare.20078996.v2.
